# The effect of a low-carbohydrate, ketogenic diet versus a low-glycemic index diet on glycemic control in type 2 diabetes mellitus

**DOI:** 10.1186/1743-7075-5-36

**Published:** 2008-12-19

**Authors:** Eric C Westman, William S Yancy, John C Mavropoulos, Megan Marquart, Jennifer R McDuffie

**Affiliations:** 1 Department of Medicine, Duke University Medical Center, Durham, NC, USA; 2 Center for Health Services Research in Primary Care, Department of Veterans' Affairs Medical Center, Durham, NC, USA

## Abstract

**Objective:**

Dietary carbohydrate is the major determinant of postprandial glucose levels, and several clinical studies have shown that low-carbohydrate diets improve glycemic control. In this study, we tested the hypothesis that a diet lower in carbohydrate would lead to greater improvement in glycemic control over a 24-week period in patients with obesity and type 2 diabetes mellitus.

**Research design and methods:**

Eighty-four community volunteers with obesity and type 2 diabetes were randomized to either a low-carbohydrate, ketogenic diet (<20 g of carbohydrate daily; LCKD) or a low-glycemic, reduced-calorie diet (500 kcal/day deficit from weight maintenance diet; LGID). Both groups received group meetings, nutritional supplementation, and an exercise recommendation. The main outcome was glycemic control, measured by hemoglobin A_1c_.

**Results:**

Forty-nine (58.3%) participants completed the study. Both interventions led to improvements in hemoglobin A_1c_, fasting glucose, fasting insulin, and weight loss. The LCKD group had greater improvements in hemoglobin A_1c _(-1.5% vs. -0.5%, p = 0.03), body weight (-11.1 kg vs. -6.9 kg, p = 0.008), and high density lipoprotein cholesterol (+5.6 mg/dL vs. 0 mg/dL, p < 0.001) compared to the LGID group. Diabetes medications were reduced or eliminated in 95.2% of LCKD vs. 62% of LGID participants (p < 0.01).

**Conclusion:**

Dietary modification led to improvements in glycemic control and medication reduction/elimination in motivated volunteers with type 2 diabetes. The diet lower in carbohydrate led to greater improvements in glycemic control, and more frequent medication reduction/elimination than the low glycemic index diet. Lifestyle modification using low carbohydrate interventions is effective for improving and reversing type 2 diabetes.

## Background

The dietary macronutrient that raises postprandial serum glucose and insulin most potently is carbohydrate [1]. This observation led to the use of diets low in carbohydrate for the treatment of diabetes before insulin or other medication therapies were available [2]. In like fashion, individuals who are insulin-deficient are instructed to estimate the amount of carbohydrate in the meal and then to administer the insulin dosage based upon the amount of dietary carbohydrate. This strong relationship between dietary carbohydrate and postprandial serum glucose led to the development of medications that block carbohydrate absorption for the treatment of type 2 diabetes [3].

Clinical studies that have lowered the percentage of dietary carbohydrate and/or the glycemic index of the carbohydrate have consistently shown improvements in glycemic control among individuals with type 2 diabetes [4-8]. In randomized studies, low-carbohydrate diets have been found effective for the treatment of obesity for durations up to 24 months [9]. While glycemic control was not a primary outcome, some of these studies additionally demonstrated improvement in glycemic parameters when carbohydrate intake was lowered. In the Nurse's Health Study cohort study, low-glycemic load diets were found to be associated with lower cardiac risk over a 20 year period [10]. One mechanism to explain these findings is that when patients are instructed to limit carbohydrate intake to low levels without mention of caloric intake, there is an overall reduction in caloric intake [11].

In several recent studies, in the outpatient setting and metabolic ward, low-carbohydrate ketogenic diets led to improvements in glycemic control among patients with diabetes [12-16]. While it may be intuitive that a low-carbohydrate ketogenic diet with fewer than 20 grams of carbohydrate intake per day would lead to better glycemic control than a "low-glycemic diet", we are not aware that this idea has been actually tested. In the present study, our hypothesis was that a diet lower in carbohydrate would lead to greater improvement in glycemic control in patients with obesity and type 2 diabetes mellitus over 24 weeks in the outpatient setting.

## Methods

### Participants

Participants were recruited from the community by newspaper advertisements. After telephone screening, potential participants were scheduled for a "screening visit" which included informed consent approved by the local institutional review board, a medical history, physical examination and laboratory tests. The inclusion criteria were: diagnosis of type 2 diabetes mellitus > 1 year (confirmed by hemoglobin A_1c _> 6.0%), onset of diabetes after age 15 years, no history of diabetic ketoacidosis, age 18–65 years old, body mass index (BMI) from 27–50 kg/m^2^, and desire to lose weight. Exclusion criteria were: unstable or serious medical condition; significant co-morbid illnesses such as liver disease (AST or ALT > 100 IU/L), kidney disease (serum creatinine > 1.5 mg/dL), cancer; pregnancy; or nursing mothers. No monetary incentives were given.

### Interventions

If study criteria were met, participants were randomized to one of two treatment groups stratified upon BMI greater or less than 32 kg/m^2 ^using a computer-generated list, and invited to attend the "baseline visit." (Measurements taken at the "screening visit" were used as the initial value in comparison testing for laboratory tests; measurements from the "baseline visit" were used as the initial value for other outcomes.) The intervention for both groups included group sessions, diet instruction, nutritional supplements, and an exercise recommendation. Group meetings took place at an outpatient research clinic every week for 3 months, then every other week for 3 months. If a participant was taking medication for diabetes or hypertension, a physician reviewed the blood glucose and blood pressure readings and made medication changes according to a pre-specified algorithm. Participants were encouraged to exercise for 30 minutes at least 3 times per week, but no formal exercise program was provided. Both groups received the same nutritional supplements known to have a mild lowering effect on blood glucose levels (vanadyl sulfate 200 mcg/day, chromium dicotinate glycinate 600 mcg/day, alpha-lipoic acid 200 mg/day) [17,18].

#### Low-carbohydrate, Ketogenic Diet Group Intervention (LCKD)

Using a lay-press diet book and additional handouts, a registered dietitian instructed participants to restrict intake of dietary carbohydrate to fewer than 20 grams per day, without explicitly restricting caloric intake [19]. Allowed foods were unlimited amounts of animal foods (i.e., meat, chicken, turkey, other fowl, fish, shellfish) and eggs; limited amounts of hard cheese (e.g., cheddar or swiss, 4 ounces per day), fresh cheese (e.g., cottage or ricotta, 2 ounces per day), salad vegetables (2 cupfuls per day), and non-starchy vegetables (1 cupful per day). Participants were encouraged to drink at least 6 glasses of permitted fluids daily. Drinking bouillon dissolved in water was recommended 2–3 times a day during the first two weeks to reduce possible side effects.

#### Low-glycemic index diet group intervention (LGID)

Using a lay-press diet book and additional handouts, a registered dietitian instructed participants to follow a low-glycemic index, reduced-calorie diet with approximately 55% of daily caloric intake from carbohydrate [20]. The energy intake was individualized to be 2.1 MJ (500 kcal) less than the participant's calculated energy intake for weight maintenance (21.6*lean body mass + 370 kcal + activity factor) [21].

### Primary outcome measure

#### Hemoglobin A_1c_

Hemoglobin A_1c _was measured at baseline, week 12, and week 24. The primary outcome was change in hemoglobin A_1c _from baseline to week 24, using an immunoassay technique. The hemoglobin A_1c _provides an estimate of glycemic control for the previous 3-month period and is predictive of clinical outcomes [22].

### Other outcome measures

#### Diet composition

All participants completed food records (5 consecutive days, including a weekend) at baseline, and during the intervention (weeks 4, 12, and 24). Participants were instructed how to document food record information and given handouts with examples of how to complete the records. A sample of completers (n = 8 for low-carbohydrate diet group; n = 7 for low-glycemic diet group) was selected for food record analysis based upon record detail. A registered dietitian analyzed the food records using a nutrition software program (Nutritionist Five, Version 1.6, First DataBank Inc., San Bruno, CA). Food record results were averaged over weeks 4, 12, and 24.

#### Vital signs

Wearing light clothing and no shoes, participants were weighed at each visit on the same calibrated scale. Body mass index was calculated as: (body weight in kilograms)/(height in meters)^2^. Systolic and diastolic blood pressures were measured in the non-dominant arm using an automated digital cuff (model HEM-725C, Omron Corp., Vernon Hills, IL) after sitting for 3 minutes. Two measurements were taken per visit and averaged for the analysis.

#### Other metabolic effects

Blood tests were obtained in the morning after at least 8 hours of fasting and processed by a commercial laboratory (Labcorp, Burlington NC). Glomerular filtration rate was estimated by using an equation containing the variables age, gender, race, and serum albumin, creatinine, and blood urea nitrogen (Modification of Diet in Renal Disease (MDRD) Study equation) [23]. Twenty-four hour urine collections for protein were collected at baseline and at 24 weeks.

#### Adverse effects

At all return visits, participants completed an open-ended side effects questionnaire. To enhance the description of side effects, participants completed a checklist of side effects commonly mentioned during weight loss studies at both the 20 and 24-week visit. These two measures were combined to report the proportion in each group who experienced an adverse effect at any time during the study.

#### Medication changes

At baseline and at all return visits, participants recorded all of their current medications with dosages and schedules.

#### Adherence

Adherence with the diet and exercise recommendations was measured by self-report, food records, and urinary ketones [24,25]. The delivery of the intervention and the assessment of outcomes were not blinded to the treatment assignment.

### Statistical analysis

For categorical outcomes, comparisons between groups were performed using the chi square test or Fisher's exact test, as appropriate. For all continuous outcomes, comparisons were made using a t-test or Wilcoxon rank-sum test as appropriate, testing the difference between groups for the change from baseline to week 24. For the primary outcome variable, a completer's analysis and last observation carried forward (LOCF) were performed, and a multiple linear regression analysis adjusting for weight change was performed to determine if the change in hemoglobin A_1c _was independent of weight loss. A p value of ≤ 0.05 was considered statistically significant. Analyses were performed using SAS Statistical Software, Version 8.02 (SAS Institute Inc., Cary, NC). In order to detect a clinically meaningful change in hemoglobin A_1c _(absolute change of 1%, SD = 1.5) with 80% power (two-sided alpha of .05) in a completers analysis, a total of 60 participants was required. To protect for dropouts, 97 participants were recruited.

### Role of the funding source

The investigators conducted the study independently of the funding source. The funding source had no involvement in conduct of the study.

## Results

### Participants

213 potential participants were screened for eligibility, and 97 were randomized. Ten participants of 48 randomized to the LCKD group, and 3 of 49 participants randomized to the LGID group discontinued the study prior to the Week 0 visit and did not receive instruction, leaving 38 in the LCKD group and 46 in the LGID group for the analyses. For the LCKD group, 21 (55.3%) completed the study; reasons for discontinuation were: 3 refused assigned diet, 2 were unsatisfied with the diet, 2 were lost to follow-up, 2 were too busy, 1 relocated, and 7 cited no reason. For the LGID group 29 (63.0%) completed the study; reasons for discontinuation were: 1 refused assigned diet, 1 was unsatisfied with the diet, 2 were lost to follow-up, 3 were too busy, 1 relocated, 1 had difficulty adhering to the diet and 9 cited no reason. The baseline characteristics of study participants are shown in Table 1. There were no clinically significant differences between the treatment groups.

**Table 1:** Baseline participant characteristics*

Characteristic	Low -glycemic, reduced-calorie diet			Low-carbohydrate, ketogenic diet		
	Enrollees (*n *= 46)	Completers (*n *= 29)	Non-completers (*n *= 17)	Enrollees (*n *= 38)	Completers (*n *= 21)	Non-completers (*n *= 17)
Age, years	51.8 ± 7.8	50.0 ± 8.4	54.9 ± 5.7	51.8 ± 7.3	51.2 ± 6.1	52.4 ± 8.7
Female gender, %	80.4	79.3	82.3	76.3	66.7	88.2
White race, %	45.7	44.8	47.1	57.9	66.7	47.1
African-American race, %	50	51.7	47.1	36.8	23.8	52.9
College degree, %	58.7	68.9	41.2	57.9	61.9	52.9
Body weight, kg	106.3 ± 20.1	105.2 ± 19.8	108.1 ± 20.9	105.5 ± 19.5	108.4 ± 20.5	101.9 ± 18.1
Body mass index, kg/m^2^	38.5 ± 5.6	37.9 ± 6.0	39.4 ± 5.0	37.7 ± 6.1	37.8 ± 6.7	37.6 ± 5.3

* Values with plus/minus signs are means ± SD.

### Hemoglobin A_1c_

**Figure 1:**
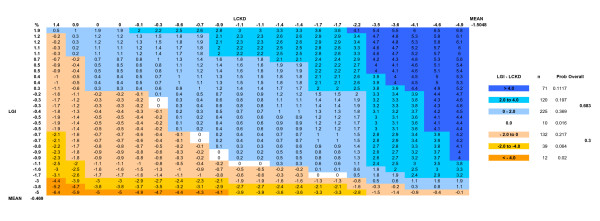
**Payoff matrix for dietary comparisons**. Matrices show the theoretical paired comparison between the change in hemoglobin A1c for each individual in the LGI group compared with each individual in the LCKD group. In rank order across the top of the matrix, the change in hemoglobin A1c from baseline to week 24 is shown for the LCKD group; down the matrix side is shown the LGI group. Each matrix element shows the difference between the value for the LGI (row) and the LCKD (column) individual (LGI-LCKD). Positive values indicate greater reduction in hemoglobin A1c for LCKD, negative values indicate greater reduction in hemoglobin A1c for LGI. At the right of the Figure, the number of matrix elements in each category are divided by the total number of matrix elements (paired differences). LGI = Low glycemic index group, LCKD = Low carbohydrate ketogenic diet group, Prob = Probability.

**Figure 2:**
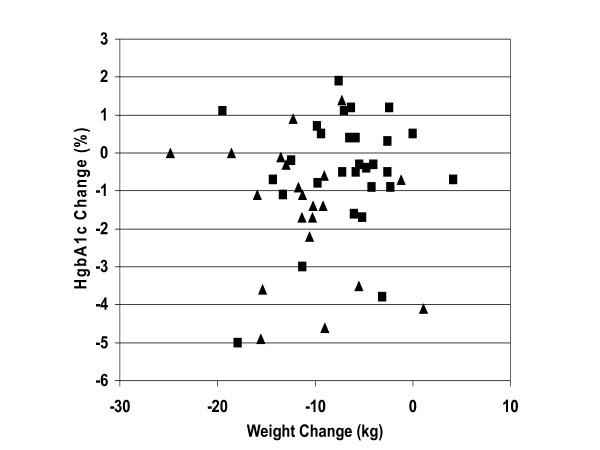
**Relationship between change in hemoglobin A1c and change in weight**. This figure plots the change in hemoglobin A1c vs. the change in weight from baseline to week 24 for each individual (r = 0.09425, p = 0.5150). The LCKD group is shown as triangles; the LGID group is shown as squares.

From baseline to 24 weeks, the reduction of mean ± SD hemoglobin A_1c _was greater for the LCKD group (8.8 ± 1.8% to 7.3 ± 1.5%, p = 0.009, within group change, n = 21) than for the LGID group (8.3 ± 1.9% to 7.8 ± 2.1% p = NS, within group change, n = 29; between groups comparison p = 0.03) (Table 2). The mean change in hemoglobin A_1c _for the LCKD group was -1.5% (95% CI: -2.30, -0.71), and for the LGID group was -0.5% (95%CI: -1.04, 0.10). Using a theoretical probability matrix comparing the change in hemoglobin A_1c _for each individual in one group to each individual in the other group, the probability of having a greater improvement in hemoglobin A_1c _was 0.683 for being assigned to the LCKD group, compared to 0.300 for being in the LGID group (Figure 1) [26]. Fasting blood glucose and insulin improved similarly for both groups over the 24 weeks. In the LOCF analysis, the mean hemoglobin A_1c _at baseline and week 24 was 8.5% and 7.5% for the LCKD group, and 8.3% and 8.0% for the LGID group (p = 0.02, between groups comparison). In a multivariate linear regression model adjusting for weight change or BMI change, the between group comparison in change in hemoglobin A_1c _approached statistical significance (p = 0.06). Additionally, there was no correlation between change in hemoglobin A_1c _and change in weight (Figure 2).

**Table 2:** Effect of diet programs on indices of glycemic control and body weight

	Week 0	Week 12	Week 24	Week 0 to 24	Between Groups	Between Groups Adjusted*
	*mean *± *sd*	*mean *± *sd*	*mean *± *sd*	*mean change*	*p value*	*p value*
LGID	*n *= 29	*n *= 29	*n *= 29			
Hemoglobin A_1c_, %	8.3 ± 1.9	7.5 ± 1.7	7.8 ± 2.1	-0.5	0.03	0.06
Fasting glucose, mg/dL	166.8 ± 63.7	140.7 ± 39.9	150.8 ± 47.4	-16.0**	0.67	0.76
Fasting insulin, μU/mL	14.8 ± 6.9	13.9 ± 9.9	12.6 ± 6.5	-2.2**	0.10	0.84
Body mass index, kg/m^2^	37.9 ± 6.0	36.5 ± 5.7	35.2 ± 6.1	-2.7**	0.05	0.10
Body weight, kg	105.2 ± 19.8	101.0 ± 16.9	98.3 ± 20.3	-6.9**	0.008	0.01
LCKD	*n *= 21	*n *= 21	*n *= 21			
Hemoglobin A_1c_, %	8.8 ± 1.8	7.2 ± 1.2	7.3 ± 1.5	-1.5**		
Fasting glucose, mg/dL	178.1 ± 72.9	156.4 ± 50.7	158.2 ± 50.0	-19.9**		
Fasting insulin, uU/mL	20.4 ± 9.3	14.3 ± 8.3	14.4 ± 6.9	-6.0**		
Body mass index, kg/m^2^	37.8 ± 6.7	34.4 ± 5.6	33.9 ± 5.8	-3.9**		
Body weight, kg	108.4 ± 20.5	100.1 ± 17.8	97.3 ± 17.6	-11.1**		

LGID = low-glycemic, reduced-calorie diet; LCKD = low-carbohydrate, ketogenic diet

* Adjusted for baseline values.

** p < 0.05 for within-group change from Baseline to Week 24.

### Medication changes

At baseline, 22 (75.9%) of the LGID group were taking hypoglycemic medications (insulin only n = 3, oral agents only n = 19), and 20 (95.2%) of the LCKD group were taking hypoglycemic medications (insulin + oral agents n = 4, insulin only n = 4, oral agents only n = 12). Twenty of 21 (95.2%) LCKD group participants had an elimination or reduction in medication, compared with 18 of 29 (62.1%) LGID group participants (p < 0.01). Table 3 shows the changes in medication for those patients who were taking insulin at baseline. Five individuals (4 in the LCKD group, 1 in the LGID group) who were taking over 20 units of insulin at baseline were no longer taking insulin at the end of the study.

**Table 3:** Changes in medication among patients taking insulin at baseline

Participant	Week 0: Total daily dose	Week 24: Total daily dose
Low-glycemic, reduced-calorie diet group (total n = 29)		
1	insulin 24 units	none
	insulin sliding scale three times a day	none
		
2	insulin 85 units	insulin 13 units
3	insulin 160 units	insulin 120 units
Low-carbohydrate, ketogenic diet group (total n = 21)		
1	insulin 50 units	none
2	insulin 90 units	none
3	insulin 32 units	none
	rosiglitizone 4 mg/metformin 2000 mg	metformin 1000 mg
		
4	insulin 40 units	none
	metformin 2000 mg	metformin 2000 mg
		
5	insulin 40 units	insulin 35 units
	insulin sliding scale three times a day	none
		
6	insulin 120 units	insulin 90 units
	metformin 2000 mg	metformin 2000 mg
		
7	insulin 135 units	insulin 60 units
8	insulin 80 units	insulin 8 units
	pioglitazone 45 mg	pioglitazone 45 mg
	glimiperide 8 mg	glimiperide 8 mg

*Medications were adjusted using a pre-specified algorithm based upon home and return visit blood capillary glucose measurements.

### Adherence

Prior to the study intervention, the mean ± SD dietary intake for both groups was 2128 ± 993 kcal, 245 ± 136 g of carbohydrate (46% of daily energy intake), 86 ± 33 g of protein (18% of daily energy intake), 88 ± 57 g of fat (36% of daily energy intake). Over the 24-week duration of the intervention, the LCKD group consumed 1550 ± 440 kcal per day, 49 ± 33 g of carbohydrate (13% of daily energy intake), 108 ± 33 g of protein (28% of daily energy intake), 101 ± 35 g of fat (59% of daily energy intake). In comparison, the LGID group consumed 1335 ± 372 kcal per day, 149 ± 46 g of carbohydrate (44% of daily energy intake), 67 ± 20 g of protein (20% of daily energy intake), 55 ± 23 g of fat (36% of daily energy intake). There was no difference in self-reported exercise between the groups: the mean number of exercise sessions per week increased from 2.0 ± 2.0 to 3.0 ± 2.0 for the LCKD group and from 2.2 ± 2.2 to 3.8 ± 2.9 for the LGID group (p = 0.39 for comparison).

### Vital signs

There was significantly greater weight loss for the LCKD than the LGID group over the 24 weeks: body weight decreased from 108.4 ± 20.5 kg to 97.3 ± 17.6 kg for the LCKD group, and from 105.2 ± 19.8 to 98.3 ± 20.3 kg for the LGID group (Table 2). Both groups had reductions in systolic blood pressure and diastolic blood pressure (Table 4).

**Table 4:** Effect of diet programs on metabolic syndrome parameters and fasting lipid profiles

	Low glycemic, reduced-calorie diet group (n = 29)			Low carbohydrate, ketogenic diet group (n = 21)		
Test	Week 0	Week 24	Week 0 to 24	Week 0	Week 24	Week 0 to 24

	*mean *± *sd*	*mean *± *sd*	*mean change*	*mean *± *sd*	*mean *± *sd*	*mean change*

Fasting glucose, mg/dL	166.8 ± 63.7	150.8 ± 47.4	-16.0 *	178.1 ± 72.9	158.2 ± 50.0	-19.9*
Waist circumference, inches	47.0 ± 5.1	42.4 ± 5.5	-4.6 *	47.1 ± 5.5	41.8 ± 5.3	-5.3 *
Triglycerides, mg/dL	167.1 ± 125.7	147.8 ± 128.5	-19.3	210.4 ± 10.3	142.9 ± 76.9	-67.5 *
HDL cholesterol, mg/dL	48.7 ± 11.8	48.7 ± 10.1	-0 ^†^	44.0 ± 8.7	49.6 ± 11.7	+5.6 * ^†^
Systolic blood pressure, mmHg	140.8 ± 15.7	130.1 ± 17.1	-10.7 *	144.4 ± 15.0	127.8 ± 13.4	-16.6 *
Diastolic blood pressure, mmHg	84.1 ± 11.0	78.5 ± 8.7	-5.6 *	83.9 ± 10.3	75.8 ± 10.9	-8.1 *
Body mass index, kg/m^2^	37.9 ± 6.0	35.2 ± 6.1	-2.7 * ^†^	37.8 ± 6.7	33.9 ± 5.8	-3.9 * ^†^
Total cholesterol, mg/dL	190.6 ± 43.8	184.8 ± 45.6	-5.8	191.4 ± 32.0	187.0 ± 35.8	-4.4
LDL cholesterol, mg/dL	113.8 ± 40.9	111.0 ± 42.2	-2.8	105.8 ± 25.7	107.1 ± 26.3	+1.3
VLDL cholesterol, mg/dL	27.7 ± 13.2	24.4 ± 12.3	-3.3*	37.3 ± 14.9	27.3 ± 15.2	-10.0*
Total cholesterol/HDL cholesterol ratio	4.1 ± 1.3	3.9 ± 1.2	-0.2	4.5 ± 1.1	4.1 ± 4.1	-0.4
Triglyceride/HDL cholesterol ratio	3.9 ± 3.7	3.3 ± 3.1	-0.6	5.2 ± 3.4	3.4 ± 3.0	-1.8*

These changes were observed with a reduction or elimination of diabetic medication as shown in Table 3.

HDL = high-density lipoprotein; LDL = low-density lipoprotein; VLDL = very-low-density lipoprotein

* p < 0.05 for within-group change from Baseline to Week 24.

^† ^p < 0.05, for between-groups comparison of changes from Baseline to Week 24.

P values with adjustment for baseline values: fasting glucose: 0.76, waist circumference: 0.43, triglycerides: 0.17, HDL: 0.09, systolic blood pressure: 0.89, diastolic blood pressure: 0.48, body mass index: 0.10, total cholesterol: 0.85, LDL: 0.79, VLDL: 0.24, total cholesterol/HDL ratio: 0.92, triglyceride/HDL ratio: 0.54.

### Other metabolic effects

For fasting lipid profiles, the LCKD group had an increase in HDL cholesterol (+12.7%), while the LGID group had no change over the 24 weeks. All 7 parameters associated with the metabolic syndrome showed improvement for the LCKD group; 5 of 7 improved for the LGID group (Table 4).

In terms of renal function, serum creatinine and calculated GFR did not change significantly over the 24 weeks for either group. There was a greater reduction in 24-hour urine protein for the LCKD group (baseline = 445 ± 1175 mg/24 hour, week 24 = 296 ± 750 mg/24 hours, n = 18), as compared with the LGID group (baseline = 276 ± 705 mg/24 hour, week 24 = 223 ± 623 mg/24 hours, n = 24, p = 0.007 for between-groups comparison).

### Adverse effects

There were no statistically significant differences between groups in reported symptomatic adverse effects. The most common symptoms experienced at any point during the study were headache (LCKD: 53.1%, LGID: 46.3%), constipation (LCKD: 53.1%, LGID: 39.0%), diarrhea (LCKD: 40.6%, LGID: 36.6%), insomnia (LCKD: 31.2%, LGID: 19.5%), and back pain (LCKD: 34.4%, LGID: 39.0%) (p > 0.05 for all comparisons).

## Discussion

In this study, both a low-glycemic index, reduced-calorie diet and a low-carbohydrate, ketogenic diet led to improvement in glycemic control, diabetic medication elimination/reduction, and weight loss in adherent overweight and obese individuals with type 2 diabetes over a 24-week period. The diet containing fewer carbohydrates, the LCKD, was most effective for improving glycemic control. In patients taking insulin, the effects were often quite powerful. For example, participants taking from 40 to 90 units of insulin before the study were able to eliminate their insulin use, while also improving glycemic control. Because this effect occurs immediately upon implementing the dietary changes, individuals with type 2 diabetes who are unable to adjust their own medication or self-monitor their blood glucose should not make these dietary changes unless under close medical supervision.

A low-carbohydrate, ketogenic diet combines two approaches that, on their own, improve blood glucose control: weight loss and a reduced-glycemic index diet. Weight loss via dietary modification has a beneficial effect on diabetes [27,28]. A reduced-glycemic index diet without weight loss can also lead to improvement in diabetic control, with the magnitude of effect of a 0.43% reduction in hemoglobin A_1c_, when compared with higher-glycemic diets of similar carbohydrate content [4]. The greater effect of the low-carbohydrate, ketogenic diet in this study appeared to be due to the lower carbohydrate intake, because statistical significance remained after adjustment for weight loss. Because "low-glycemic" diets in previous studies typically contain from 40–60% of calories from carbohydrate, it is possible that the beneficial effect of "low-glycemic" diets could be augmented by further reduction of the absolute amount of carbohydrate, or by a reduction in caloric content.

While this study was a treatment trial of individuals with type 2 diabetes, lifestyle modification has been shown to prevent type 2 diabetes in the Diabetes Prevention Program (DPP). The intensive lifestyle modification arm of the DPP included a calorie- and fat-restricted diet with an energy intake of 1380 kcal/day for women and 1583 kcal/day for men, and a percentage of energy from carbohydrate of 54% [29]. While the effect was stronger than medication, the intensive lifestyle group developed diabetes at a rate of 20% after 4 years. Future research should include the use of lower-carbohydrate diets for the treatment and prevention of type 2 diabetes.

Like previous studies, we found that the LCKD led to weight reduction, improvement in glycemic control, and elevation in HDL-cholesterol, but no deterioration in fasting lipid parameters. Extending these findings, we observed that all metabolic syndrome components were improved by the LCKD [30]. It is interesting to note that the LGID group reported consuming fewer calories than the LCKD group, yet had less weight loss. This may reflect problems with the diet data as collected, issues with differential physical activity, or metabolic inefficiency (leading to increased energy expenditure) which may occur during the consumption of a carbohydrate-restricted diet.

Limitations of this study include the lack of blinding of physicians and outcome assessors to treatment group, and the use of food records. The study participants were community volunteers, and predominantly women, which may limit generalization of these findings to clinical populations and men. The analysis and presentation of only detailed food records may bias the estimate of food intake. We chose the "completer analysis" as the primary outcome because we were interested in answering the question of what might be expected from patients who can adhere to the intervention. The LOCF analysis might generalize better to a population of patients who have different food preferences from their assigned diet, who lose/lack motivation, or who experience other barriers to dietary change. Another possible limitation is the baseline imbalance in the primary outcome, HgA_1c_, which occurred despite random allocation. The equation used to calculate energy requirements for the LGID participants may underestimate requirements, particularly in obese people. This would result in more severe energy restriction than the 500 kcal deficit as stated, which might bias the weight loss effects in favor of the LGID.

It is often presumed that obesity is the cause of type 2 diabetes, but there are clearly instances where obesity occurs without type 2 diabetes, and instances where type 2 diabetes occurs without obesity. In this study, the change in hemoglobin A1c was independent of the change in weight (Figure 2). This supports the concept that weight change and glycemic control are not serially linked but rather may be the result of the same pathophysiologic process, such as abnormal insulin metabolism.

The underlying principle of carbohydrate-restriction and the historic precedents of using the low-carbohydrate diet for type 2 diabetes suggest that the low-carbohydrate approach may be one of the most effective dietary treatments for diabetes. Our findings support this position, and it suggests that the burden of proof be placed upon alternative points of view. The dearth of randomized, controlled trials using the low-carbohydrate approach for type 2 diabetes, despite the historical and current clinical use of these approaches, challenges the idea that the randomized controlled trial should be the only guide of scientific inquiry and clinical practice.

## Conclusion

In summary, lifestyle modification using two diets that reduce carbohydrate intake led to improvement in glycemic control, diabetic medication elimination/reduction, and weight loss in overweight and obese individuals with type 2 diabetes over a 24-week period in the outpatient setting. The diet containing fewer carbohydrates, the low-carbohydrate, ketogenic diet, was more effective for improving glycemic control than the low glycemic diet. Lifestyle modification using low-carbohydrate diet interventions are effective for improving obesity and type 2 diabetes, and may play an important role in reversing the current epidemic of 'diabesity.'

## Competing interests

The authors declare that they have no competing interests.

## Authors' contributions

EW and WY designed the study. EW, WY, JM, and MM assisted in data collection and analysis. EW performed most of the data analysis and drafted the first manuscript. All authors participated in revising the manuscript, and read and approved the final manuscript.
